# Comparing the Costs and Diagnostic Outcomes of Replacing Cytology with the QIAsure DNA Methylation Test as a Triage within HPV Primary Cervical Cancer Screening in The Netherlands

**DOI:** 10.3390/diagnostics13243612

**Published:** 2023-12-06

**Authors:** Krishnan Puri Sudhir, Eva Kagenaar, Michelle Meijer, Albertus T. Hesselink, Elisabeth Adams, Katy M. E. Turner, Susie Huntington

**Affiliations:** 1 Aquarius Population Health, Unit 29 Tileyard Studios, London N7 9AH, UK; 2 Self-Screen B.V., Plesmanlaan 125, 1066 CX Amsterdam, The Netherlands

**Keywords:** human papillomavirus infections, early detection of cancer, cost–benefit analysis, uterine cervical neoplasms, DNA methylation, The Netherlands

## Abstract

Detecting hypermethylation of tumour suppressor genes could provide an alternative to liquid-based cytology (LBC) triage within HPV primary cervical screening. The impact of using the QIAsure^®^ FAM19A4/mir124-2 DNA Methylation Test (QIAGEN, N.V, Hilden, Germany) on CIN3+ diagnoses, retention, unnecessary colposcopies, and programme costs is unknown. A decision-tree model was developed to compare LBC with the QIAsure Methylation testing to guide colposcopy referral. Incorporating clinician- and self-sampling pathways the model was informed by the Dutch cervical cancer screening programme, published studies, and manufacturer data. Clinical and cost outcomes were assessed using two scenarios for DNA methylation testing and LBC relative performance. Sensitivity analyses (deterministic and probabilistic) were performed to assess model and parameter uncertainty. A range of self-sampling uptake was assessed in scenario analyses. For the screening cohort (*n* = 807,269) where 22.1% self-sampled, the number of unnecessary colposcopies and CIN3+ diagnoses varied according to the relative performance of methylation testing and LBC. Irrespective of relative performance, the cost per complete screen was lower and fewer people were lost to follow-up when using DNA methylation testing. The results indicate that, within an HPV primary screening programme that incorporates self-sampling, using the QIAsure Methylation Test for triage reduces the cost per screen compared to LBC.

## 1. Introduction

Cervical cancer is one of the leading causes of mortality globally but can be prevented by national screening programmes [1,2]. In the Netherlands, women and people with a cervix aged 30–65 are eligible to participate in HPV primary cervical screening [3,4,5]. The Netherlands was the first country to introduce self-sampling as part of the screening programme in 2017. Initially, it was offered only to those declining the initial invite to screen via clinician-collected sampling. Since 2022, it has been offered as an alternative option in the initial invite [6]. Within the screening pathway, samples are first tested for the presence of high-risk HPV genotypes (hrHPV); positive samples are then triaged using liquid-based cytology (LBC) [5]. People with an LBC result that indicates cytological abnormalities are referred for colposcopy. During colposcopy, those with grade 2/3 cervical intraepithelial neoplasia (CIN2+) are treated to prevent progression to cervical cancer [5].

There are challenges in using LBC within screening programmes [7]. Performing LBC is resource intensive and the number of trained cytologists is in decline [8]. Test performance varies and its low specificity results in unnecessary referral for colposcopy and other interventions [9,10,11]. Further, as an increasing number of HPV-vaccinated people enter the screening cohort, HPV prevalence is expected to fall, which will further reduce test specificity for LBC [12,13]. In addition, LBC requires a clinician-collected sample (i.e., cells collected from the cervix) to ensure the accurate detection of abnormal cells [14]. Therefore, people whose self-collected sample is hrHPV-positive must attend an additional appointment to have a clinician-collected sample taken for LBC. This additional step in the self-sampling pathway increases sample collection costs and the risk of loss to follow-up (LTFU) in hrHPV-positive women, who are at higher risk of cervical cancer than hrHPV-negative women. As the use of self-sampling increases over time in the Netherlands and elsewhere [15], minimising loss to follow-up for attending cytology within screening is an increasingly important consideration. 

Hypermethylation of host tumour suppressor genes is an indication of oncogenesis [16,17]. DNA methylation assays such as the QIAsure Methylation Test^®^ (QIAGEN, N.V, Hilden, Germany), which detects promotor hypermethylation of the genes FAM19A4 and miR124-2 and is available as a clinically validated CE-IVD commercial assay, could be used instead of LBC for triage in national screening programmes to guide colposcopy referral and can be used on self-collected samples. 

This study aims to assess the impact on screening costs and CIN diagnoses of using the QIAsure Methylation Test in place of LBC to guide colposcopy referral within HPV primary cervical cancer screening in the Netherlands. In the main analysis, two scenarios are assessed using different relative performance of DNA methylation testing and LBC. Different ratios of self-sampling and clinician-collected sampling uptake were explored in additional scenario analyses. The results can inform decision-making about the future use of DNA methylation tests for triage within HPV primary cervical screening programmes. 

## 2. Materials and Methods

### 2.1. Model Type and Structure

**Figure 1:**
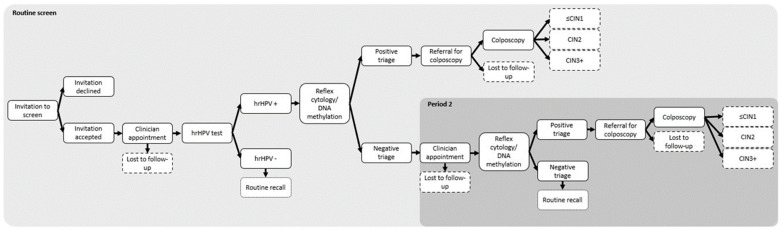
Screening pathway for clinician-collected sampling for cervical cancer screening in The Netherlands. Footnote: Figure 1 represents clinician-collected sampling pathways for standard of care (cytology) and DNA methylation. Self-sampling pathways are presented in Supplementary Figures S1 and S2.

A cost–consequence analysis from a health system perspective was performed to compare using LBC (standard of care (SoC)) with using the QIAsure *FAM19A4/mir124-2* DNA Methylation Test (QIAGEN, N.V, Hilden, Germany) for triage to guide colposcopy referral following an hrHPV-positive result. A decision tree model was constructed in Excel v2210 (Microsoft, Redmond, WA, USA) to simulate a single screening cohort. The model’s structure was informed by the protocol used in the Dutch Cervical Screening Programme. Both clinician- and self-sampling (i.e., vaginal swab used at home and mailed to the laboratory) pathways were incorporated. Figure 1 illustrates the pathway for clinician-collected sampling for both triage strategies (self-sampling pathways presented in Supplementary Figures S1 and S2). Screening data from 2021 were used to inform the proportions of clinician- (77.9%) and self-sampling (22.1%) in the base case [18]. Key model assumptions are listed in Supplementary Table S1. Model endpoints were discharge to routine screening, loss to follow-up, or CIN diagnosis following colposcopy.

### 2.2. Time Horizon

The model simulated one complete screening cycle which included the routine screen and early recall periods. ‘Routine screen’ included the invite to screen, sample collection, hrHPV testing, LBC/methylation, and colposcopy (where indicated). For those who were hrHPV-positive but LBC/methylation-negative at routine screen, the early recall period included sample collection, six-month early recall for LBC/DNA methylation testing, and colposcopy (where indicated). Longer-term costs and outcomes were not considered.

### 2.3. Outcomes 

The primary outcomes assessed were the cost per complete screen, number of unnecessary colposcopies (i.e., resulting in ≤CIN1 finding), number of CIN3+ diagnoses, and loss to follow-up. A complete screen refers to either an hrHPV-negative result at routine screen, normal LBC/negative methylation result at early recall, or completed colposcopy at routine screen or early recall. 

The secondary outcomes assessed were the total number of complete screens, total screening costs, number of CIN2+ diagnoses, cost per CIN2+ diagnosis, and cost per CIN3+ diagnosis. Each outcome was calculated for the complete screening cycle. The difference between outcome values for LBC and methylation, and the percentage change were also calculated. 

### 2.4. Population

The model simulated a screening cohort of 807,629 individuals representing the number of people screening each year, using data from 2019 [19], i.e., before COVID-19 pandemic disruptions. 

### 2.5. Cost Inputs

The following costs were included in the model: clinician appointment for sample collection, self-sampling kit (including postage), hrHPV laboratory testing, LBC, methylation testing, organisational costs for the screening programme, and colposcopy appointments (Table 1). Costs associated with sample collection, LBC, and hrHPV test differed depending on the sample collection method and whether this was for the routine screen or early recall [20]. An additional EUR 12,705,838 was included to reflect the organisational costs of the screening programme (reported as EUR 18.50 per analysed hrHPV sample) [20]. The average reported costs for colposcopy procedures were used, excluding the 10% lowest and highest values [21]. Prices were inflated to 2022 where required using consumer price indices reported by the International Monetary Fund [22]. Costs were not discounted as the model’s time horizon was less than one year. Indirect costs were not considered since a health system perspective was employed.

The cost for LBC was informed by publicly available subsidy scheme data published by the Dutch Ministry of Health, Welfare, and Sport [20]. The estimated cost of the QIAsure Methylation Test (QIAGEN, N.V, Hilden, Germany) was provided by the manufacturer and staff costs for performing the assay were calculated (Supplementary Materials, Table S5). 

### 2.6. Clinical and Disease Detection Inputs

Data from 2019 were used to inform overall screening uptake, colposcopy referrals, loss to follow-up, and CIN outcomes following colposcopy for routine screening (categorised as ≤1, 2, 3+) since 2020–2022 were affected by the COVID-19 pandemic and are unlikely to represent future numbers [19]. Data from 2021 were used to inform the uptake of clinician- and self-sampling since the proportion of people self-sampling is anticipated to remain at this level or increase in the future. The proportion of CIN2 and CIN3+ at routine screen was used for the proportion at early recall (Supplementary Materials, Table S9) since early recall data were not available. 

The HPV prevalence and the distribution of LBC results and CIN outcomes in women who did not screen or were lost to follow-up were assumed to be the same as in women who did screen, since no data were available on these. 

No data were available directly comparing the test performance of LBC and QIAsure Methylation Test^®^ (QIAGEN, N.V, Hilden, Germany) in a screening population reflecting the Dutch national screening programme. To reflect the range of possibilities, two scenarios were assessed (see Supplementary Section S4 for rationale and test performance data). In Scenario 1, LBC had a higher sensitivity for CIN2 and CIN3+ and a higher specificity compared to methylation, with absolute performance informed by data from Bonde et al. [23] (Supplementary Table S7). In Scenario 2, methylation testing had a higher sensitivity for CIN2 and CIN3+ and a higher specificity compared to LBC, with absolute performance informed by a prospective study of *FAM19A4* methylation (Luttmer et al.) [24] (Supplementary Table S8).

For both scenarios, the sensitivity and specificity of LBC were used to back-calculate the ‘true disease states’ of the screening cohort. The sensitivity and specificity of methylation testing were then used to calculate the anticipated diagnostic outcomes for the methylation strategy (see Supplementary Materials, Section S4). 

### 2.7. Uncertainty Analyses 

#### 2.7.1. Deterministic Sensitivity Analysis (DSA)

A one-way deterministic sensitivity analysis was performed to evaluate the impact of varying each input parameter for three outcomes: cost per complete screen, number of unnecessary colposcopies (defined as ≤CIN1 diagnoses), and number of CIN3+ diagnoses. Low and high values for the deterministic sensitivity analysis were informed by surveillance data. In the absence of this, ±10% or ±20% of the baseline value was used. The difference in outcomes for the standard of care and methylation strategies was calculated for all simulations. 

#### 2.7.2. Probabilistic Sensitivity Analysis (PSA)

Probabilistic sensitivity analysis was performed to investigate the robustness of results given a wide range of plausible inputs. Outcomes assessed for the probabilistic sensitivity analysis were the same as for the deterministic sensitivity analysis. Each input parameter was assigned an estimated statistical distribution (Table 2), a beta distribution for probabilities, and a gamma distribution for costs, as per standard recommendations. Probabilistic sensitivity analysis simulations were run using the Monte Carlo method with 1000 iterations and 95% credible intervals (CIs) were estimated for each of the outputs considered.

#### 2.7.3. Scenario Analyses 

In scenario analyses, the uptake of self-sampling was varied between 0% and 100% in 25% increments to reflect a variety of possibilities. In addition, a scenario analysis was performed where the sensitivity and specificity of methylation testing and LBC were identical. 

## 3. Results

### 3.1. Base-Case Results 

Table 2 presents the primary and secondary outcomes for the LBC and methylation screening strategies and the difference between outcomes for the two scenarios assessed.

#### 3.1.1. Base-Case Results for Scenario 1 (where LBC Has Higher Sensitivity and Specificity than Methylation Testing)

The average cost per complete screen was EUR 98.10 for the methylation strategy and EUR 98.92 for the LBC strategy, an incremental difference of EUR −0.82 (95% CI EUR −1.78 to EUR 0.15) representing a 0.8% reduction in costs for methylation compared to LBC. For the cohort of 807,269 women, the total screening costs were EUR 40,895,452 for the methylation strategy and EUR 41,099,596 for the LBC strategy, representing a 0.5% reduction in cost for methylation compared to LBC. The methylation strategy resulted in more unnecessary colposcopies (≤CIN1 diagnoses) (5746 compared to 4369 for LBC) and fewer CIN3+ diagnoses (2233 compared to 2470 for LBC). Fewer women were lost to follow-up in the methylation strategy (20.8%, *n* = 1405) (5361 for methylation and 6766 for LBC).

#### 3.1.2. Base-Case Results for Scenario 2 (Methylation Testing Has Higher Sensitivity and Specificity than LBC)

The average cost per complete screen was EUR 104.07 for the methylation strategy and EUR 107.90 for the LBC strategy, and the incremental cost per screen was EUR −3.83 (95% CI EUR −4.80 to EUR 2.77) representing a 3.6% reduction in costs for methylation compared to LBC. The total screening costs were EUR 43,212,775 for the methylation strategy and EUR 44,547,572 for the LBC strategy, representing a 3.0% reduction in cost for methylation compared to LBC. The methylation strategy resulted in 1191 fewer unnecessary colposcopies (6690 compared to 7880 for LBC) and 475 additional CIN3+ diagnoses (5169 compared to 4693 for LBC). As with Scenario 1, 2385 fewer women (25.4%) were lost to follow-up in the methylation strategy (7004 compared to 9389 for LBC). 

### 3.2. Sensitivity Analyses 

#### 3.2.1. Deterministic Sensitivity Analysis

Deterministic sensitivity analyses were performed separately for Scenarios 1 and 2. Parameters with the most effect on the cost per complete screen, number of unnecessary colposcopies, and number of CIN3+ diagnoses are presented as tornado plots (Supplementary Materials, Figure S3). While all model inputs were varied in the deterministic sensitivity analysis, only the five parameters with the most effect on outcomes are presented in the tornado plots. For Scenario 1, the most impactful input variables on cost per complete screen were the cost of methylation testing, the chance of hypermethylation positivity (for the methylation strategy), or the proportion with abnormal LBC findings (for the LBC strategy) at routine screen on clinician-collected hrHPV-positive samples. These same variables were also impactful on the cost per complete screen for Scenario 2.

#### 3.2.2. Probabilistic Sensitivity Analysis 

Results from the probabilistic sensitivity analysis are presented as scatter plots (Supplementary Materials, Figure S4) and were used to calculate the 95% credible intervals presented in Table 2.

### 3.3. Scenario Analyses

All results for the scenarios assessed are presented in Table 3. Using data for Scenario 1, with 100% clinician-collected sampling, the incremental cost per complete screen was EUR −0.76. The DNA methylation strategy resulted in 29 more lost to follow-up, 1327 more unnecessary colposcopies, and 357 fewer CIN3+ diagnoses compared to LBC. When assessing the other extreme (100% of people self-sampling), the incremental cost per screen was EUR −0.77, while the methylation strategy resulted in 6462 fewer people lost to follow-up and 188 more CIN3+ diagnoses compared to the LBC strategy. 

When the performance of methylation testing was assumed to be identical to that of LBC in Scenario 1.6 (informed by LBC performance data in Scenario 1)*,* the incremental cost per screen was EUR −1.04, while the methylation strategy resulted in 1487 fewer lost to follow-up, 142 additional unnecessary colposcopies, and 131 more CIN3+ diagnoses.

Using performance data from Scenario 2 and assuming 100% clinician-collected sampling, the result for the incremental (methylation vs. LBC) cost per complete screen was EUR −4.31. The methylation strategy resulted in 1043 fewer lost to follow-up, 1562 fewer unnecessary colposcopies, and 217 additional CIN3+ diagnoses compared to LBC. When assessing the other extreme (100% self-sampling), the incremental cost per screen was EUR −2.02, while the methylation strategy resulted in 7116 fewer lost to follow-up, 118 fewer unnecessary colposcopies, and 1387 additional CIN3+ diagnoses. 

When the test performance of methylation testing was identical to LBC (Scenario 2.6) (informed by LBC performance data in Scenario 2) the incremental cost per screen was EUR −0.24, and the methylation strategy resulted in 1329 fewer LTFU, 279 additional unnecessary colposcopies, and 257 additional CIN3+ diagnoses. 

## 4. Discussion

### 4.1. Main Findings 

The model simulated the use of the QIAsure *FAM19A4/miR124-2* Methylation Test (QIAGEN, N.V, Hilden, Germany) in place of LBC for triage to inform referral to colposcopy within an HPV primary screening pathway, where clinician-collected and self-sampling are used. The methylation test strategy resulted in a lower cost per screen and less loss to follow-up in both scenarios, i.e., when methylation testing was assumed to have higher sensitivity and specificity than LBC and vice versa. The methylation strategy resulted in fewer unnecessary colposcopies and more CIN3+ diagnoses in the scenario where methylation testing had a higher sensitivity and specificity than LBC (Scenario 2). The opposite was true in Scenario 1, where LBC had a higher sensitivity and specificity.

### 4.2. Strengths and Limitations

This is the first study to model the costs and diagnostic outcomes of using the QIAsure DNA Methylation Test (QIAGEN, N.V, Hilden, Germany) for triage in a national cervical cancer screening programme. The findings can be used to inform decision-making about screening protocols in the Netherlands and similar settings. 

A key limitation of the model is the lack of data available to inform the relative test performance of LBC and methylation testing. LBC is to some extent subjective, and sensitivity and specificity vary widely between settings [11]. Consequently, although methylation testing is a standardised method, its performance relative to LBC varies. The few studies which have compared the test performance used study populations and protocols which do not reflect the Dutch national screening population or programme [17,25,26]. To reflect the uncertainty about the relative performance, two scenarios were assessed. Scenario 1 was informed by data from a multicentre retrospective study assessing QIAsure *FAM19A4/miR124-2* Methylation Test and LBC performance on hrHPV-positive cervical scrapes from women in Slovenia, Denmark, Scotland, and the Netherlands [23]. In this study, the sensitivity and specificity of LBC were higher than observed in the Dutch screening programme and treatment was guided by the LBC result, introducing a bias for LBC which may explain the lower relative performance of methylation testing (Supplemental Material Table S8). Scenario 2 was informed by a prospective multicentre cohort assessing the performance of LBC and methylation of *FAM19A4* in hrHPV-positive cervical scrapes from women attending gynaecology outpatient clinics in the Netherlands [24]. The sensitivity and specificity of LBC are lower than in Scenario 1. Although the sensitivity of methylation testing for CIN3+ is higher than in Scenario 1, the specificity is lower (Supplemental Materials, Table S8).

LBC and methylation detect different things; LBC detects visible cell changes and methylation tests detect hypermethylation of host tumour suppressor genes involved in cervical carcinogenesis. Another important related limitation is that a positive QIAsure *FAM19A4/miR124-2* Methylation Test result, where CIN2+ is the outcome (from colposcopy), is not equivalent to an ‘abnormal’ LBC result where CIN2+ is the outcome because a proportion of CIN2/3+ regress and do not result in cancer if left untreated [27,28]. In the absence of data to inform what proportion of CIN2+ would regress or progress, our model categorised all colposcopies in people with CIN2+ as ‘necessary’; however, this would not be the case if the triage test could accurately identify cases that will progress [24,25]. Early studies suggest that methylation of *FAM19A4/miR124-2* predicts progression to cervical cancer and that methylation testing can better distinguish lesions that will progress to cancer [26]. Women with a CIN2+ outcome (in colposcopy) who get a negative QIAsure *FAM19A4/miR124-2* Methylation Test result are more likely to regress (to ≤CIN1) than those with a positive test result [26]. This means that it may be feasible to safely extend the recall screening period for women who are hrHPV-positive and methylation test negative, which would reduce screening costs. Longer-term clinical data are needed to inform the modelling of this. 

As with all models, some assumptions were required. The test performance of DNA methylation was assumed to be the same for self- and clinician-collected samples. Although there is some evidence indicating that the sensitivity of methylation testing in hrHPV-positive women is higher in self-collected samples [29], there were insufficient data to inform the relative test performance of LBC *vs*. methylation testing in different sample types. Consequently, the DNA methylation test performance might be underestimated for the self-sampling pathway. 

Secondly, the prevalence of CIN 1, 2, and 3+ was assumed to be the same in people attending colposcopy as in those lost to follow-up. Previous research has shown that disease prevalence, including the prevalence of cervical cancer, is typically higher in non-attenders [30,31,32]. Assuming the same distribution of CIN grades among those lost to follow-up is therefore likely to underestimate the benefit of CIN3+ detection in the methylation strategy. 

Thirdly, the percentage of people lost to follow-up was assumed to remain unchanged after the reporting of the surveillance data (15 months after invitations were sent). This likely results in an overestimation of loss to follow-up, particularly for the recall screening group, since women might not have had the opportunity to schedule an early recall appointment. 

Lastly, an average cost for colposcopy was used, i.e., independent of the CIN outcome. In reality, the cost of colposcopy may be higher for more advanced disease states since performing a biopsy can make the procedure last longer, require additional consultations, and incur pathology costs. 

Where possible, published cost data were used but costs associated with laboratory testing could be affected by several factors including improved automation and the use of computer-assisted reading of LBC slides. The model did not consider differences in the rates of invalid results or the turnaround time to deliver results, metrics which would be influenced by many factors and likely change if DNA methylation testing were routinely used at scale.

Many of the input variables were informed using pre-COVID-19 screening data. Several changes were made in 2022 to the screening eligibility criteria, colposcopy referral criteria, and early recall period [6] (Supplementary Materials, Section S6). However, no screening outcome data following these changes were available when developing the model. Notably, in 2022, the offer of self-screening was made available at the initial invite to screen rather than as a secondary option to non-responders. The impact of this and the changes planned for 2023 on the overall screening uptake are unclear but it is anticipated the proportion of people self-sampling will increase. Our scenario analyses (Table 3) indicate that this would further reduce loss to follow-up and decrease the cost per screen. 

### 4.3. Interpretation

The model results indicate that methylation testing could provide an efficient and less costly option than LBC in screening programmes where self-sampling is available. The results are of use in many settings including in countries without an established cervical cancer screening programme, in settings where clinician-collected sampling is challenging, or where there is poor LBC performance or limited availability of LBC (Scenarios 1.7 and 2.7). In countries with an established HPV primary cervical screening programme where self-sampling is used, LBC is a common triage strategy [33]. In this context, DNA methylation testing would eliminate the need for an additional clinician-collected smear, reducing loss to follow-up and costs. The results of the scenario analysis could inform decision-making in other settings. Future research should consider estimating the effects on disease outcomes as hrHPV and CIN prevalence are likely to differ across contexts. 

DNA methylation assays could have additional benefits, not captured in our model. For example, DNA methylation assays could improve patient experience and acceptability of screening because they remove the need to attend a sample collection appointment following an hrHPV-positive self-sample. The use of methylation assays can be automated and thus could improve laboratory workflow. Less specialist training is needed to perform the assay which could be particularly beneficial in contexts where there are insufficient or declining numbers of cytologists. 

### 4.4. Future Research

Our model reflects the cervical cancer screening pathway used in the Netherlands and is informed using surveillance data. Recent changes have been made to the screening protocol and further changes are planned (Supplementary Materials: Section S6). HPV genotyping has recently been introduced; for women with hrHPV genotypes 16/18, there is a lower cytology threshold for colposcopy referral than for women with other hrHPV genotypes. Since screening data reflecting these changes is not yet available, genotyping was not incorporated into our model. Using methylation as a triage marker for HPV-positive women, as stand-alone or in combination with other markers (e.g., genotyping), in screening could allow for more efficient triage algorithms compared to current practice [34,35] and be used to better inform surveillance intervals. A longer-term model informed by clinical data would be required to assess the impact of such strategies on clinical outcomes and cost-effectiveness.

The recent increase in research on DNA methylation to detect cervical cancer [17,23,25] suggests that there is interest in its potential use for triage within national cervical screening programmes, especially where self-sampling is offered. However, additional evidence is required on the test performance of LBC and DNA methylation assays on different sample types (including urine), and their relative test performance in groups that reflect screening populations. Furthermore, our model only reflects one strategy for using methylation testing for triage. 

## 5. Conclusions

The per screen cost does not pose a barrier to the introduction of QIAsure DNA Methylation Test (QIAGEN, N.V, Hilden, Germany) for triage within the Dutch cervical cancer screening programme since it is lower than the per screen cost of using LBC irrespective of which data are used to inform the relative performance of these two tests. In addition, using DNA methylation testing for triage could have benefits for laboratory workflow, and for patient experience and avoiding loss to follow-up for HPV self-sampling users.

## Figures and Tables

**Table 1:** Model parameters to compare QIAsure Methylation Test^®^ (QIAGEN, N.V, Hilden, Germany) and cytology in the cervical cancer screening programme in the Netherlands.

Parameter	Strategy	Base Value (EUR)	Low Value (EUR) ^1^	High Value (EUR) ^1^	PSA Distribution	Reference/ Comment
**Cost data**						
*Clinician-collected sampling*						
Sample collection (routine screen ^2^)	Both ^4^	24.63	22.17	27.09	Gamma	[20]
Sample collection (early recall ^3^)	Both	21.37	19.23	23.51	Gamma	[20]
hrHPV testing	Both	16.57	14.91	18.23	Gamma	[20]
LBC (routine screen ^2^)	LBC	26.99	24.29	29.69	Gamma	[20]
LBC (early recall ^3^)	LBC	34.92	31.43	38.41	Gamma	[20]
*Self-sampling*						
Self-sampling kit	Both	10.98	9.88	12.08	Gamma	[20]
Sample collection (early recall ^3^)	Both	20.08	18.07	22.09	Gamma	[20]
hrHPV testing	Both	15.16	13.64	16.68	Gamma	[20]
LBC (routine screen ^2^)	LBC	43.92	39.53	48.31	Gamma	[20]
LBC (early recall ^3^)	LBC	34.92	31.43	38.41	Gamma	[20]
*All sampling strategies*						
Colposcopy ^5^	Both	545.00	490.50	599.50	Gamma	[21]
Organisational costs ^6^	Both	10.93	14.79	9.93	Gamma	Supplementary Table S6
DNA methylation	DNA methylation	29.44	27.02	42.02	Gamma	Estimate, Supplementary Table S5
**Probabilities using annual surveillance data** [18,19]						
*Clinician-collected sampling*						
Clinician-collected sampling	Both	77.90%	80.11%	75.69%	Beta	[18]
Attends sample collection appointment	Both	91.39%	82.25%	100.00%	Beta	[19]
hrHPV-positive result	Both	9.96%	8.97%	10.96%	Beta	[19]
LTFU at colposcopy referral (routine screen ^2^)	Both	28.51%	35.66%	21.36%	Beta	[19]
LTFU at colposcopy referral (early recall ^3^)	Both	42.52%	51.15%	33.90%	Beta	[19]
LTFU at early recall (early recall ^3^)	Both	3.44%	0.00%	3.78%	Beta	[19]
*Self-sampling*						
hrHPV-positive result	Both	8.38%	7.54%	9.22%	Beta	[19]
Returns for sample collection appointment	LBC	79.25%	71.33%	87.18%	Beta	[19]
LTFU at colposcopy referral (routine screen ^2^)	Both	20.75%	14.52%	26.97%	Beta	[19]
LTFU at colposcopy referral (early recall ^3^)	Both	76.19%	78.57%	40.48%	Beta	[19]
LTFU at early recall (early recall ^3^)	Both	3.47%	0.00%	3.81%	Beta	[19]
**Scenario 1: LBC has higher sensitivity and specificity than DNA methylation testing** [23]						
*Clinician-collected sampling*						
LBC abnormal (routine screen ^2^)	LBC	28.33%	25.50%	31.17%	Beta	Supplementary Table S15
LBC abnormal (early recall ^3^)	LBC	7.18%	6.47%	7.90%	Beta	Supplementary Table S15
DNA methylation positive (routine screen ^2^)	DNA methylation	28.57%	25.72%	31.43%	Beta	Supplementary Table S15
DNA methylation positive (early recall ^3^)	DNA methylation	7.25%	6.52%	7.97%	Beta	Supplementary Table S15
*Self-sampling*						
LBC abnormal (routine screen ^2^)	LBC	30.16%	27.14%	33.18%	Beta	Supplementary Table S15
LBC abnormal (early recall ^3^)	LBC	2.60%	2.34%	2.86%	Beta	Supplementary Table S15
DNA methylation positive (routine screen ^2^)	DNA methylation	32.20%	28.98%	35.42%	Beta	Supplementary Table S15
DNA methylation positive (early recall ^3^	DNA methylation	2.78%	2.50%	3.05%	Beta	Supplementary Table S15
**Scenario 2 DNA methylation testing has higher sensitivity and specificity than LBC** ^7^ [24]						
*Clinician-collected sampling*						
LBC abnormal (routine screen ^2^)	LBC	56.78%	51.10%	62.46%	Beta	Supplementary Table S15
LBC abnormal (early recall ^3^)	LBC	7.18%	6.47%	7.90%	Beta	Supplementary Table S15
DNA methylation positive (routine screen ^2^)	DNA methylation	46.61%	41.95%	51.27%	Beta	Supplementary Table S15
DNA methylation positive (early recall ^3^)	DNA methylation	5.90%	5.31%	6.49%	Beta	Supplementary Table S15
*Self-sampling*						
LBC abnormal (routine screen ^2^)	LBC	60.16%	54.15%	66.18%	Beta	Supplementary Table S15
LBC abnormal (early recall ^3^)	LBC	2.60%	2.34%	2.86%	Beta	Supplementary Table S15
DNA methylation positive (routine screen ^2^)	DNA methylation	51.04%	45.93%	56.14%	Beta	Supplementary Table S15
DNA methylation positive (early recall ^3^)	DNA methylation	2.21%	1.99%	2.43%	Beta	Supplementary Table S15

^1^ Low and high values are informed by routine surveillance data; in the absence of such data, ±10% or ±20% of the baseline value is used. ^2^ Indicates the routine screen attended. ^3^ Indicates early recall at 6 months following routine screen. ^4^ Indicates the parameter is used in both LBC and DNA methylation pathways. ^5^ Includes booking, staff time, colposcopy procedure, and consumables. ^6^ Operational costs incurred in delivering screening programme. ^7^ Only parameters that change values when switching test performance data from Bonde et al. [9] to Luttmer et al. [24] have been reported. hrHPV, high-risk human papillomavirus; LBC, liquid-based cytology, LTFU, lost to follow-up; PSA, probabilistic sensitivity analysis.

**Table 2:** Baseline results for a cohort of 807,629 women aged 30 to 60 screened for cervical cancer using HPV primary pathway with either LBC or DNA methylation triage to inform referral for colposcopy.

Outcome	LBC	DNA Methylation	% Change ^1^	Incremental Difference ^2^ (95% CI) *
**Scenario 1: base-case results**				
Number of complete screens	415,483	416,888	0.3%	1405
Number LTFU	6766	5361	−20.8%	−1405
Number of ≤CIN1 diagnoses	4369	5746	31.5%	1376 (1208; 1551)
Number of CIN2+ diagnoses	4369	3509	−19.7%	−860
Number of CIN3+ diagnoses	2470	2233	−9.6%	−236 (−353; −117)
Total screening costs (EUR)	41,099,596	40,895,452	−0.5%	−204,144
Costs related to sample collection (EUR) ^3^	9,738,670	9,338,105	−4.1%	−400,565
Costs related to laboratory testing (EUR) ^4^	8,946,292	8,861,200	−1.0%	−85,092
Costs related to colposcopy (EUR) ^5^	4,762,298	5,043,811	5.9%	281,513
Cost per complete screen (EUR) ^6^	98.92	98.10	−0.8%	−0.82 (−1.78; 0.15)
Cost per CIN2+ diagnosis (EUR) ^7^	9407	11,654	23.9%	2247
Cost per CIN3+ diagnosis (EUR) ^8^	16,642	18,312	10.0%	1671
**Scenario 2: base-case results**				
Number of complete screens	412,859	415,244	0.6%	2385
Number LTFU	9389	7004	−25.4%	−2385
Number of ≤CIN1 diagnoses	7880	6690	−15.1%	−1191 (−1409; −929)
Number of CIN2+ diagnoses	8294	7345	−11.4%	−949
Number of CIN3+ diagnoses	4693	5169	10.1%	475 (275; 713)
Total screening costs (EUR)	44,547,572	43,212,775	−3.0%	−1,334,797
Costs related to sample collection (EUR) ^3^	9,509,079	9,259,968	−2.6%	−249,111
Costs related to laboratory testing (EUR) ^4^	8,571,124	8,651,684	0.9%	80,560
Costs related to colposcopy (EUR) ^5^	8,815,033	7,648,787	−13.2%	−1,166,247
Cost per complete screen (EUR) ^6^	107.90	104.07	−3.6%	−3.83 (−4.80; 2.77)
Cost per CIN2+ diagnosis (EUR) ^7^	5371	5883	9.5%	512
Cost per CIN3+ diagnosis (EUR) ^8^	9492	8361	−11.9%	−1131

^1^ The difference between LBC and DNA methylation as a percentage of the value for LBC value. ^2^ The difference between outcome values for LBC and DNA methylation. ^3^ Includes costs for clinician-collected sample collection, self-sampling kits, and kit postage. ^4^ Includes costs for laboratory hrHPV testing, cytology, and DNA methylation. ^5^ Includes costs for colposcopy procedure. ^6^ Calculated as the total cost of screening divided by the number of complete screens. ^7^ Calculated as the total cost of screening divided by the number of CIN2+ diagnoses. ^8^ Calculated as the total cost of screening divided by the number of CIN3+ diagnoses. * Credible intervals of 95% are calculated in the PSA. CIN, cervical intraepithelial neoplasia; LBC, liquid-based cytology; LTFU, lost to follow-up refers to those who receive a positive hrHPV or cytology result but do not attend for further investigation and those who do not attend the early recall at period 2. Scenario 1 is informed using performance data from Bonde et al. [23] and Scenario 2 using performance data from Luttmer et al. [24].

**Table 3:** Results for additional scenarios varying uptake of sampling methods and triage test performance for a cohort of 807,629 women aged 30 to 60 screened for cervical cancer using HPV primary pathway.

Scenario	Number of People LTFU	Number of ≤CIN1 Diagnoses	Number of CIN3+ Diagnoses	Total Cost of Screening (EUR)	Cost per Complete Screen (EUR)
**Scenario 1: LBC has higher sensitivity and specificity than DNA methylation testing** [23]					
Scenario 1.1: 100% clinician-collected sampling, 0% self-sampling					
LBC	5215	4913	2530	42,603,221	104.31
DNA methylation test	5245	6240	2173	42,289,928	103.55
Incremental ^1^	29	1327	−357	−313,293	−0.76
Scenario 1.2: 75% clinician-collected sampling, 25% self-sampling					
LBC	10,477	3068	2326	37,499,862	86.73
DNA methylation test	5638	4562	2378	37,557,026	85.90
Incremental	−4839	1494	52	57,164	−0.83
Scenario 1.3: 50% clinician-collected sampling, 50% self-sampling					
LBC	8723	3683	2394	39,200,982	92.37
DNA methylation test	5507	5121	2309	39,134,660	91.52
Incremental	−3216	1438	−84	−66,322	−0.85
Scenario 1.4: 25% clinician-collected sampling, 75% self-sampling					
LBC	6969	4298	2462	40,902,101	98.23
DNA methylation test	5376	5681	2241	40,712,294	97.40
Incremental	−1593	1383	−221	−189,807	−0.83
Scenario 1.5: 0% clinician-collected sampling, 100% self-sampling					
LBC	12,230	2453	2258	35,798,743	81.29
DNA methylation test	5769	4002	2446	35,979,392	80.52
Incremental	−6462	1550	188	180,650	−0.77
Scenario 1.6: same test performance for LBC and DNA methylation test ^2^					
LBC	6766	4369	2470	41,099,596	98.92
DNA methylation test	5279	4511	2600	40,811,627	97.88
Incremental	−1487	142	131	−287,970	−1.04
**Scenario 2: DNA methylation testing has higher sensitivity and specificity than LBC** [24]					
Scenario 2.1: 100% clinician-collected sampling, 0% self-sampling					
LBC	7810	8748	4766	46,278,868	114.04
DNA methylation test	6766	7186	4983	44,643,326	109.72
Incremental	−1043	−1562	217	−1,635,542	−4.31
Scenario 2.2: 75% clinician-collected sampling, 25% self-sampling					
LBC	13,171	5803	4518	40,402,785	94.02
DNA methylation test	7574	5501	5612	39,787,981	91.40
Incremental	−5598	−302	1094	−614,804	−2.62
Scenario 2.3: 50% clinician-collected sampling, 50% self-sampling					
LBC	11,384	6785	4601	42,361,479	100.44
DNA methylation test	7304	6063	5402	41,406,429	97.24
Incremental	−4080	−722	802	−955,050	−3.21
Scenario 2.4: 25% clinician-collected sampling, 75% self-sampling					
LBC	9597	7766	4684	44,320,173	107.11
DNA methylation test	7035	6624	5193	43,024,878	103.34
Incremental	−2562	−1142	509	−1,295,296	−3.77
Scenario 2.5: 0% clinician-collected sampling, 100% self-sampling					
LBC	14,959	4821	4435	38,444,090	87.84
DNA methylation test	7843	4939	5821	38,169,532	85.82
Incremental	−7116	118	1387	−274,558	−2.02
Scenario 2.6: same test performance for LBC and DNA methylation ^2^					
LBC	9389	7880	4693	44,547,572	107.90
DNA methylation test	8060	8159	4950	44,591,104	107.66
Incremental	−1329	279	257	43,532	−0.24

^1^ Calculated as the difference between relevant outcomes for DNA methylation versus cytology. ^2^ Sensitivity and specificity for detecting CIN outcomes using DNA methylation on hrHPV-positive samples assumed equal to that for cytology. CIN, cervical intraepithelial neoplasia; LBC, liquid-based cytology; LTFU, lost to follow-up (refers to those who receive a positive hrHPV or cytology result but do not attend for further investigation and those who do not attend the early recall screen).

## Data Availability

Data are contained within the article and Supplementary Materials.

## Supplementary Materials

The following supporting information can be downloaded at: https://www.mdpi.com/article/10.3390/diagnostics13243612/s1. Reference [36] was cited in the Supplementary Materials.

## References

[1] Sung H., Ferlay J., Siegel R.L., Laversanne M., Soerjomataram I., Jemal A., Bray F. (2021). Global Cancer Statistics 2020: GLOBOCAN Estimates of Incidence and Mortality Worldwide for 36 Cancers in 185 Countries. CA Cancer J. Clin..

[2] Crosbie E.J., Einstein M.H., Franceschi S., Kitchener H.C. (2013). Human papillomavirus and cervical cancer. Lancet.

[3] Chrysostomou C.A., Stylianou C.D., Constantinidou A., Kostrikis G.L. (2018). Cervical Cancer Screening Programs in Europe: The Transition Towards HPV Vaccination and Population-Based HPV Testing. Viruses.

[4] Sawaya G.F., Smith-McCune K., Kuppermann M. (2019). Cervical Cancer Screening: More Choices in 2019. JAMA.

[5] The National Institute for Public Health and Environment Framework for the Execution of Cervical Cancer Population Screening. https://www.rivm.nl/documenten/framework-for-execution-of-cervical-cancer-population-screening.

[6] Actuele Ontwikkelingen—Bevolkingsonderzoek Baarmoederhalskanker. https://www.rivm.nl/bevolkingsonderzoek-baarmoederhalskanker/professionals/actuele-ontwikkelingen.

[7] Davies-Oliveira J., Smith M., Grover S., Canfell K., Crosbie E. (2021). Eliminating cervical cancer: Progress and challenges for high-income countries. Clin. Oncol..

[8] Gezondheidsraad (2021). Verbetermogelijkheden Bevolkingsonderzoek Baarmoederhalskanker. Den Haag. https://www.gezondheidsraad.nl/documenten/adviezen/2021/10/19/verbetermogelijkheden-bevolkingsonderzoek-baarmoederhalskanker.

[9] Nanda K., McCrory D.C., Myers E.R., Bastian L.A., Hasselblad V., Hickey J.D., Matchar D.B. (2000). Accuracy of the Papanicolaou Test in Screening for and Follow-up of Cervical Cytologic Abnormalities: A Systematic Review. Ann. Intern. Med..

[10] Fahey M.T., Irwig L., Macaskill P. (1995). Meta-analysis of Pap Test Accuracy. Am. J. Epidemiol..

[11] Palmer T.J., Nicoll S.M., McKean M.E., Park A.J., Bishop D., Baker L., Imrie J.E.A. (2013). Prospective parallel randomized trial of the MultiCyte^TM^ ThinPrep^®^ imaging system: The Scottish experience. Cytopathology.

[12] Aitken C.A., Van Agt H.M.E., Siebers A.G., Van Kemenade F.J., Niesters H.G.M., Melchers W.J.G., Vedder J.E.M., Schuurman R., Van Den Brule A.J.C., Van Der Linden H.C. (2019). Introduction of primary screening using high-risk HPV DNA detection in the Dutch cervical cancer screening programme: A population-based cohort study. BMC Med..

[13] Bergeron C., Giorgi-Rossi P., Cas F., Schiboni M.L., Ghiringhello B., Dalla Palma P., Minucci D., Rosso S., Zorzi M., Naldoni C. (2015). Informed Cytology for Triaging HPV-Positive Women: Substudy Nested in the NTCC Randomized Controlled Trial. JNCI J. Natl. Cancer Inst..

[14] Brink A.A.T.P., Meijer C.J.L.M., Wiegerinck M.A.H.M., Nieboer T.E., Kruitwagen R.F.P.M., van Kemenade F., Fransen Daalmeijer N., Hesselink A.T., Berkhof J., Snijders P.J.F. (2006). High concordance of results of testing for human papillomavirus in cervicovaginal samples collected by two methods, with comparison of a novel self-sampling device to a conventional endocervical brush. J. Clin. Microbiol..

[15] Serrano B., Ibáñez R., Robles C., Peremiquel-Trillas P., de Sanjosé S., Bruni L. (2022). Worldwide use of HPV self-sampling for cervical cancer screening. Prev. Med..

[16] De Strooper L.M.A., Hesselink A.T., Berkhof J., Meijer C.J.L.M., Snijders P.J.F., Steenbergen R.D.M., Heideman D.A.M. (2014). Combined CADM1/MAL methylation and cytology testing for colposcopy triage of high-risk HPV-positive women. Cancer Epidemiol. Biomark. Prev..

[17] De Strooper L.M.A., Berkhof J., Steenbergen R.D.M., Lissenberg-Witte B.I., Snijders P.J.F., Meijer C.J.L.M., Heideman D.A.M. (2018). Cervical cancer risk in HPV-positive women after a negative *FAM19A4/mir124-2* methylation test: A post hoc analysis in the POBASCAM trial with 14 year follow-up. Int. J. Cancer.

[18] IKNL (2022). Monitor Bevolkingsonderzoek Baarmoederhalskanker 2021. https://www.rivm.nl/documenten/monitor-bevolkingsonderzoek-baarmoederhalskanker-2021.

[19] IKNL (2021). Monitor Bevolkingsonderzoek Baarmoederhalskanker 2019. https://www.rivm.nl/documenten/monitor-bevolkingsonderzoek-baarmoederhalskanker-2019.

[20] The Minister of Health, Welfare and Sport, The Netherlands. Subsidieregeling Publieke Gezondheid (Public Health Subsidy Regulations). https://wetten.overheid.nl/BWBR0018743/2019-01-01/#HoofdstukII_Paragraaf2_Artikel46.

[21] Nederlandse Zorg Autoriteit Open Data van de Nederlandse Zorgautoriteit. https://www.opendisdata.nlt.

[22] IMF Data. https://data.imf.org/regular.aspx?key=61015892.

[23] Bonde J., Floore A., Ejegod D., Vink F.J., Hesselink A., van de Ven P.M., Valenčak A.O., Pedersen H., Doorn S., Quint W.G. (2021). Methylation markers FAM19A4 and miR124-2 as triage strategy for primary human papillomavirus screen positive women: A large European multicenter study. Int. J. Cancer.

[24] Luttmer R., De Strooper L.M.A., Berkhof J., Snijders P.J.F., Dijkstra M.G., Uijterwaal M.H., Steenbergen R.D.M., van Kemenade F.J., Rozendaal L., Helmerhorst T.J.M. (2016). Comparing the performance of FAM19A4 methylation analysis, cytology and HPV16/18 genotyping for the detection of cervical (pre)cancer in high-risk HPV-positive women of a gynecologic outpatient population (COMETH study). Int. J. Cancer.

[25] Dick S., Kremer W.W., De Strooper L.M.A., Lissenberg-Witte B.I., Steenbergen R.D.M., Meijer C.J.L.M., Berkhof J., Heideman D.A.M. (2019). Long-term CIN3+ risk of HPV positive women after triage with FAM19A4/miR124-2 methylation analysis. Gynecol. Oncol..

[26] Kremer W.W., Dick S., Heideman D.A.M., Steenbergen R.D.M., Bleeker M.C.G., Verhoeve H.R., van Baal W.M., van Trommel N., Kenter G.G., Meijer C.J.L.M. (2022). Clinical Regression of High-Grade Cervical Intraepithelial Neoplasia Is Associated with Absence of FAM19A4/miR124-2 DNA Methylation (CONCERVE Study). J. Clin. Oncol..

[27] Tainio K., Athanasiou A., Tikkinen K.A.O., Aaltonen R., Hernándes J.C., Glazer-Livson S., Jakobsson M., Joronen K., Kiviharju M., Kalliala I. (2018). Clinical course of untreated cervical intraepithelial neoplasia grade 2 under active surveillance: Systematic review and meta-analysis. BMJ.

[28] McCredie M.R., Sharples K.J., Paul C., Baranyai J., Medley G., Jones R.W., Skegg D.C. (2008). Natural history of cervical neoplasia and risk of invasive cancer in women with cervical intraepithelial neoplasia 3: A retrospective cohort study. Lancet Oncol..

[29] Luttmer R., De Strooper L.M., Dijkstra M.G., Berkhof J., Snijders P.J., Steenbergen R.D., van Kemenade F.J., Rozendaal L., Helmerhorst T.J., Verheijen R.H. (2016). FAM19A4 methylation analysis in self-samples compared with cervical scrapes for detecting cervical (pre)cancer in HPV-positive women. Br. J. Cancer.

[30] Peirson L., Fitzpatrick-Lewis D., Ciliska D., Warren R. (2013). Screening for cervical cancer: A systematic review and meta-analysis. Syst. Rev..

[31] Ernstson A., Urdell A., Forslund O., Borgfeldt C. (2020). Cervical cancer prevention among long-term screening non-attendees by vaginal self-collected samples for hr-HPV mRNA detection. Infect. Agent. Cancer.

[32] Gok M., Heideman D.A.M., van Kemenade F.J., Berkhof J., Rozendaal L., Spruyt J.W.M., Voorhorst F., Belien J.A.M., Babovic M., Snijders P.J.F. (2010). HPV testing on self collected cervicovaginal lavage specimens as screening method for women who do not attend cervical screening: Cohort study. BMJ.

[33] Maver P.J., Poljak M. (2020). Primary HPV-based cervical cancer screening in Europe: Implementation status, challenges, and future plans. Clin. Microbiol. Infect..

[34] Dick S., Vink F.J., Heideman D.A.M., Lissenberg-Witte B.I., Meijer C.J.L.M., Berkhof J. (2022). Risk-stratification of HPV-positive women with low-grade cytology by FAM19A4/miR124-2 methylation and HPV genotyping. Br. J. Cancer.

[35] Polman N.J., de Haan Y., Veldhuijzen N.J., Heideman D.A.M., de Vet H.C.W., Meijer C.J.L.M., Massuger L.F.A.G., van Kemenade F.J., Berkhof J. (2019). Experience with HPV self-sampling and clinician-based sampling in women attending routine cervical screening in the Netherlands. Prev. Med..

[36] Rebolj M., Bonde J., Preisler S., Ejegod D., Rygaard C., Lynge E. (2016). Human Papillomavirus assays and cytology in primary cervical screening of women aged 30 years and above. PLoS ONE.

