# Supplementary material for: Comparing the Costs and Diagnostic Outcomes of Replacing Cytology with the QIAsure DNA Methylation Test as a Triage within HPV Primary Cervical Cancer Screening in The Netherlands
Source: Diagnostics (Basel). 2023 Dec 6;13(24):3612. doi: 10.3390/diagnostics13243612 (PMC10742725; doi:10.3390/diagnostics13243612)
Supplement: Supplementary file 1 [file diagnostics-13-03612-s001.zip › diagnostics-2720611-supplementary.pdf]

SUPPLEMENTARY MATERIAL

Comparing costs and diagnostic outcomes of replacing LBC with the QIAsure DNA methylation assay as a triage within HPV primary cervical cancer screening in the Netherlands

Section S1: Model assumptions

Table S1. Key model assumptions — Page 3

Section S2: Self-sampling pathways

Figure S1. LBC self-screening pathway — Page 4

Figure S2. DNA methylation screening pathway — Page 5

Section S3. Cost variables

Table S2. Variables used to inform the costs of clinician-collected sampling for LBC strategy — Page 6

Table S3. Variables used to inform costs of self-sampling for LBC strategy — Page 7

Table S4. Variables used to inform costs of DNA methylation — Page 8

Table S5. DNA methylation test cost — Page 8

Table S6. Organisational costs per woman invited — Page 8

Section S4. Calculating the probability of test results and CIN outcomes

Overview — Page 9

Data on the test performance of LBC and DNA methylation — Page 9

Table S7. Test performance data of LBC and QIAsure FAM19A4/miR124-2 methylation test reported by Bonde *et al.* for all four countries combined — Page 10

Table S8. Test performance data of LBC and QIAsure FAM19A4 methylation test reported by Luttmer *et al*. — Page 11

Calculating the true disease states within the screening population — Page 12

Table S9. Calculating true disease states at routine screen using LBC data and CIN outcomes reported in the cervical screening programme for clinician collected samples using Bonde *et al.* — Page 12

Table S10. Calculating true disease states at early recall screen using LBC data and CIN outcomes reported in the cervical screening programme for clinician-collected samples at period 2 and using LBC data from Bonde *et al.* — Page 14

Table S11. Calculating anticipated CIN diagnostic outcomes at routine screen for clinician collected samples using DNA methylation data from Bonde *et al.* — Page 15

Table S12. Calculating true disease states at routine screen using LBC data and CIN outcomes reported in the cervical screening programme for self-collected samples — Page 16

and using LBC data from Bonde *et al.*

Table S13. Calculating true disease states at early recall screen using LBC data and CIN outcomes reported in the cervical screening programme for self-collected samples and using LBC data from Bonde *et al.* Page 16

Table S14. Calculating anticipated CIN diagnostic outcomes at routine screen for clinician collected samples using DNA methylation data from Bonde *et al.* Page 17

Repeating both sets of calculations using Luttmer *et al.* Page 17

Table S15. Parameter data used in the model to inform the probability of a negative or positive result for LBC and DNA methylation pathways. Page 17

Table S16. Parameter data used in the model to inform the probability of CIN outcomes for LBC and DNA methylation pathways. Page 18

Section S5. Sensitivity analyses

Figure S3. DSA tornado plots of the five most impactful variables on cost per complete screen, unnecessary colposcopies, and CIN3+ diagnoses Page 19

Figure S4. PSA scatter plots of the incremental cost per screen *vs*. incremental CIN3+ diagnoses and incremental cost per screen *vs*. incremental number of unnecessary colposcopies Page 20

Section S6. The Dutch national cervical cancer screening programme Page 21

Section S1: Model Assumptions

Table S1. Key Model Assumptions

1. Colposcopy has 100% sensitivity and specificity at detecting/identifying CIN 1, 2, 3+ i.e., the outcome of the colposcopy is correct and the true disease state of the patient.
2. The prevalence of CIN1, 2 and 3+ among women who are lost to follow-up is the same as women who complete screening.
3. The proportion of CIN2 and CIN3+ in period 2 is the same as in routine screen.
4. The test performance of QIAsure DNA Methylation assay is independent of sample collection method - i.e., clinician-collected and self-collected samples.

Section S2: Self-sampling pathways

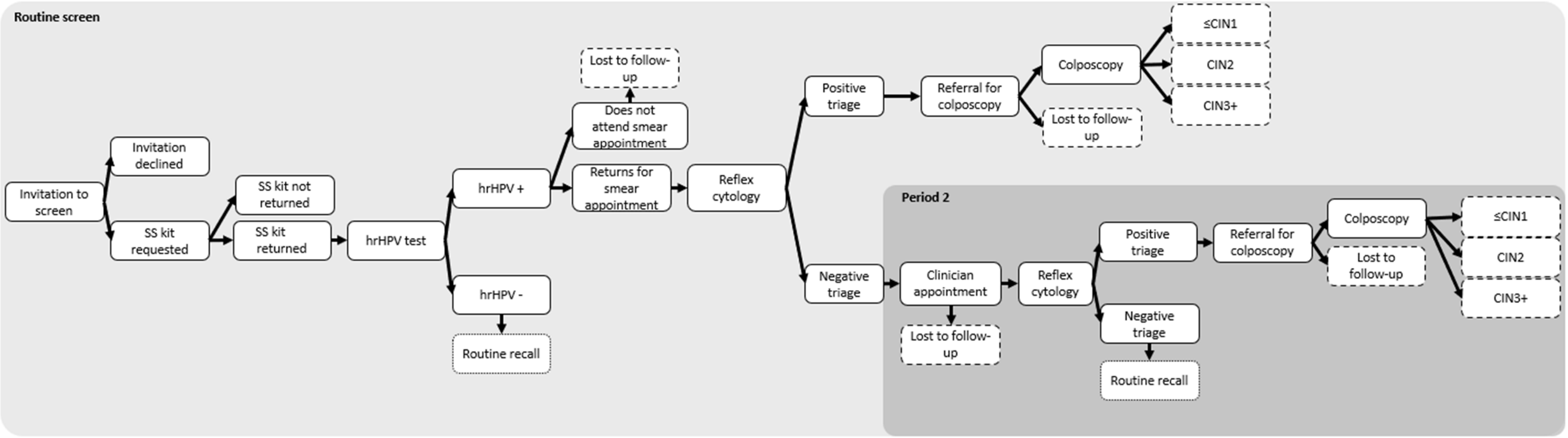

Figure S1. LBC self-screening pathway

SS, self-sampling, hrHPV, high risk human papillomavirus; DNA, Deoxyribonucleic acid; CIN, cervical intra-epithelial neoplasia

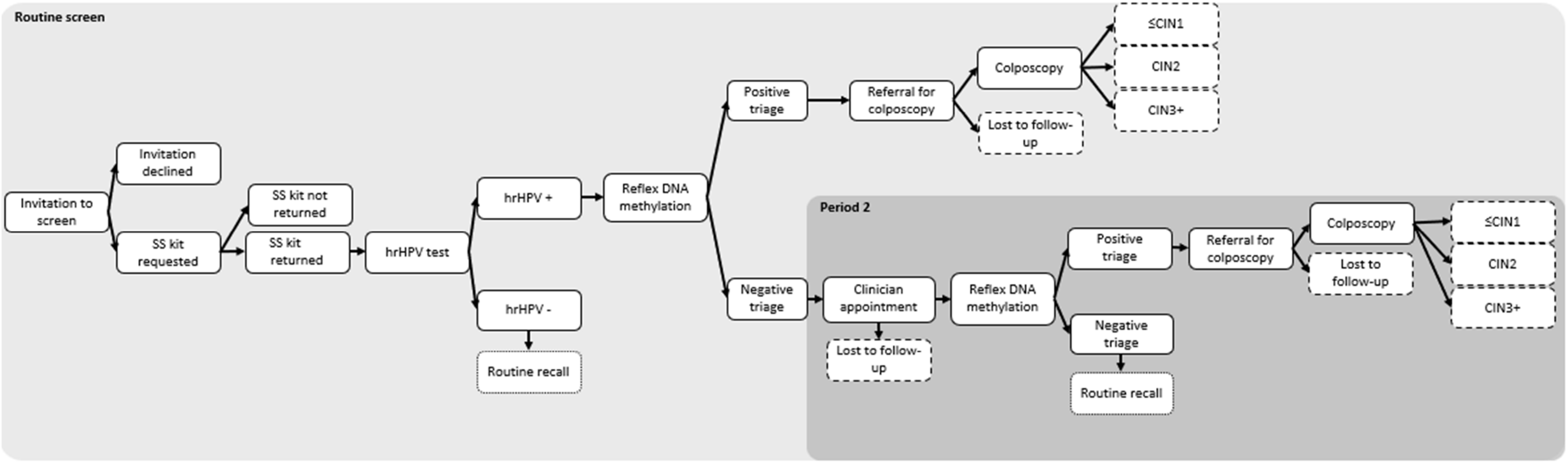

Figure S2. DNA methylation self-screening pathway

SS, self-sampling, hrHPV, high risk human papillomavirus; DNA, Deoxyribonucleic acid; CIN, cervical intra-epithelial neoplasia

Section S3. Cost variables

Table S2. Variables used to inform the costs of clinician collected sampling for the LBC strategy

Variable	Value	Comment/Reference
hrHPV testing[1]		
Sample collection	€ 24.63	Includes cost of consumables and clinician time. National Public Health Subsidy Scheme, Chapter II, Article 46 [20]
hrHPV laboratory test	€ 16.57	National Public Health Subsidy Scheme, Chapter II, Article 46 [20]
LBC testing in HPV+[1]		
Liquid based LBC[2]	€ 26.99	National Public Health Subsidy Scheme, Chapter II, Article 46 [20]
Early recall: smear and LBC	€ 56.29	National Public Health Subsidy Scheme, Chapter II, Article 46 [20]
Colposcopy[1]		
Colposcopy	€ 545.00	Includes consultation/s, outpatient clinic visits/ consumables and staff time.<br>Open data van de Nederlandse Zorgautoriteit [21].<br>Uitvoerend specialisme: 0307 – Medische specialisten, obstetrie en gynaecologie<br>Hoofd diagnosecode: G19 - Cervixafwijking incl. afwijkende cervixcytologie<br>Zorgproduct: 181105007 - Onderzoek(en) en/of behandeling(en) bij een afwijking van de baarmoederhals<br><br>Executing specialism: 0307 – Medical specialists, obstetrics and gynaecology<br>Main diagnosis code: G17 – Cervical abnormalities incl. Abnormal cervical LBC.<br>Care product: 181105007 – Examination of and/or treatment(s) for cervical abnormalities.
Other[1]		
Organisation costs	€ 19.50	National Public Health Subsidy Scheme, Chapter II, Article 46 [20]<br>Incurred per total analysed hrHPV tests either clinician-collected or self-collected.<br>Multiplied by the total number of analysed hrHPV tests in the self- and clinician-collected and added to the overall costs of each pathways.

hrHPV, high-risk human papillomavirus.

[1]Costs inflated from 2019 to 2022 where necessary.

[2]These costs have been optimized for screening through the stringent tender procedure and are for the complete LBC workflow comprising amongst other things: staff time, reagents and reading.

Table S3. Variables used to inform costs of self-sampling for LBC strategy

Variable	Value	Comment/Reference
hrHPV testing[1]		
Sample collection	€ 10.98	Self-sampling kit including postage. National Public health subsidy scheme, Chapter II, Article 46 [20]
hrHPV laboratory test	€ 15.16	National Public Health Subsidy Scheme, Chapter II, Article 46 [20]
LBC testing in HPV+[1]		
Sample collection	€ 20.08	Includes cost of self-sampling kit and postage. National Public Health Subsidy Scheme, Chapter II, Article 46 [20]
Liquid based LBC	€ 43.92	National Public Health Subsidy Scheme, Chapter II, Article 46 [20]
Colposcopy[1]		
Colposcopy: See costs for clinician collected sampling		
Other[1]		
Organisation costs: See costs for clinician collected sampling		

hrHPV, high-risk human papillomavirus; HPV+, positive human papillomavirus test.

[1]Costs inflated from 2019 to 2022 where necessary.

Table S4. Variables used to inform costs of DNA methylation

Variable	Value	Comment/Reference
Screening[1]		
Initial clinician-collected screening	€ 21.37	National Public Health Subsidy Scheme, Chapter II, Article 46 [20]<br>Calculated: Costs for clinician smear appointment – costs for liquid-based LBC
hrHPV[1]		
Laboratory costs	€ 15.16	National Public Health Subsidy Scheme, Chapter II, Article 46 [20]
Other[1]		
Organisation costs	€ 19.50	
Price of QIAsure DNA Methylation Assay	€ 27.42	Estimate informed by average from manufacturer
**Total costs**		

Footnote: hrHPV, high-risk human papillomavirus; ASP, Average selling price; LP, list price

[1]Costs inflated from 2019 to 2022 where necessary.

Table S5. DNA methylation test cost

Variable	Value		Reference/Comment
Staff time per sample	€ 2.02		
Time per sample		3.43 min	Estimate based on 240 minutes hands on time to process 70 samples plus 2 QC samples.
Labour cost		€ 0.59/min	Estimate based on €35.40 hourly cost for laboratory technician.
Assay cost	€ 27.42		
**Total cost per sample**	**€ 29.44**		

Footnote: DNA methylation is not currently used in the screening programme; therefore, published reimbursement values were not available. Therefore, the cost was estimated but may vary when used at scale. It does not include additional sample pre-treatment costs which would vary by laboratory. For this reason, the cost of DNA methylation was varied considerably in sensitivity analysis. Abbreviations: min, minutes; QC, quality control.

Table S6. Organisational costs per woman invited to screen

Variable	Value	Reference/Comment
Cost per person invited for screening		
Cost per analysed HPV test (2019)	€ 19.50	
Number of women screened (2019)	452,624	
Total cost	€ 8,826,168	Calculated as cost per analysed HPV test × women screened
Number of women invited for screening	807,629	
Cost per woman invited	€ 10.93	Calculated as total cost ÷ women invited for screening

Footnote: HPV, human papillomavirus.

Section S4. Calculating the probability of test results and CIN outcomes

Overview

The CIN outcomes for the SoC pathway were based on surveillance data from cervical screening programme in the Netherlands. For the DNA methylation pathway, CIN outcomes were calculated by using this surveillance data in combination with performance data reported by studies looking at the diagnostic results for LBC and DNA methylation for samples with a histology result.

As a first step, the performance data of LBC was used in combination with surveillance data to back calculate the true disease state of the screening population. The performance data for DNA methylation was then used in combination with the true disease state data to calculate the anticipated diagnostic outcomes for the population i.e., the number of CIN diagnoses made or missed.

Data on the test performance of LBC and DNA methylation

Limited data were available to inform the performance of LBC and DNA methylation. Furthermore, the test performance varies across the available sources. Therefore, the performance data comparing LBC and DNA methylation came from two separate studies which to our knowledge are the only available sources that report test performance of LBC and DNA methylation in the same study population.

Study 1: Bonde *et al.* [23] is a multicentre retrospective study assessing the clinical performance of the QIAsure *FAM19A4/miR124-2* methylation test. Using hrHPV positive cervical samples collected from women (≥29 years old) being screened in four different European countries: Slovenia (n=928), Denmark (n=424), Scotland (n=161) and the Netherlands (n=871). In total, 2384 samples were tested for methylation and of these, 899 had a histology result within 2 years. Different settings used different clinically validated HPV assays to determine HPV status, different liquid-based LBC (LBC) sample collection medias and DNA extraction methods.

In this study, the sensitivity for CIN3+ was 91% for LBC and 79% for DNA methylation and the specificity was 83% for LBC and 78% for DNA methylation (Table 7). A key limitation of this study is that the specificity and sensitivity of LBC is considerably higher than anticipated in other settings – and is higher than the performance reported by Luttmer *et al.* [24]. For example, in the large Horizon study [37] the specificity of LBC is 70.8%, sensitivity for CIN2+ is 66.7% and sensitivity for CIN3+ is 78.4%.

Table S7. Performance data used in Scenario 1: performance data of LBC and QIAsure *FAM19A4/miR124-2* methylation test reported by Bonde *et al.* [23] for all four countries combined

	≤CIN1		CIN2		CIN3+		Total	
**LBC outcome**								
Abnormal (positive)	302	16.8%	98	81.7%	213	91.4%	613	28.5%
Normal (negative)	1,497	83.2%	22	18.3%	20	8.6%	1,539	71.5%
Total	1,799		120		233		2,152	
**DNA methylation outcome**								
Methylation (positive)	437	21.7%	56	46.7%	183	78.5%	676	28.6%
No methylation (negative)	1,575	78.3%	64	53.3%	50	21.5%	1,689	71.4%
Total	2,012		120		233		2,365	

Footnote: LBC data extracted from, Table 3. DNA methylation data extracted from Table 2 for *FAM19A4/miR124-2* methylation test.

Study 2: Luttmer *et al.* [24] is a prospective observational multi-centre cohort study in the Netherlands. Samples were collected from women visiting a gynaecology outpatient clinic for any reason. Self-collected samples were initially used to test for hrHPV, then clinician collected samples in those who tested positive for hrHPV (n=717). In total, 556 samples were tested using LBC, FAM19A4 methylation and HPV16/18 genotyping. Although data were collected for women aged 18-70 years, only data for women >30 years were used in our calculations.

In this study, the sensitivity for CIN3+ was 85% for LBC and 88% for DNA methylation and the specificity was 58% for LBC and 66% for DNA methylation (Table 5). LBC performance is considerably lower than reported in Bonde *et al.* [23].

Table S8. Test performance data of LBC and QIAsure *FAM19A4* methylation test reported by Luttmer *et al.* [24]

	≤CIN1		CIN2		CIN3+		Total	
**LBC outcome**								
Abnormal (positive)	74	42.0%	45	88.2%	51	85.0%	170	59.2%
Normal (negative)	102	58.0%	6	11.8%	9	15.0%	117	40.8%
Total	176		51		60		287	
**DNA methylation outcome**								
Methylation (positive)	60	34.1%	26	51.0%	53	88.3%	139	48.4%
No methylation (negative)	116	65.9%	25	49.0%	7	11.7%	148	51.6%
Total	176		51		60		287	

LBC data extracted from Table 7 and Table 8. DNA methylation data extracted from Table 7 and Table 8 for FAM19A4 methylation.

Calculating the true disease states within the screening population

Cervical screening programme surveillance data (from 2019) on diagnostic outcomes in people with LBC abnormal result were used as the starting point (Table 9, gold boxes)

Of the 12,683 people with 'abnormal' LBC, the CIN outcome data was reported for 8,459 (66.7%) and therefore included in the calculations. The number of people with a 'normal' LBC result (n=28,439) was reduced by the same percentage (to become n=18,967).

Performance data from Bonde *et al.* (Table 8) for the accuracy of LBC (percentage of true negatives, true positives, false negatives and false positives) to detect CIN2/3+ was used to calculate the number of people with each CIN outcome within the LBC 'normal' group (Table 9, blue boxes), given the known number of people with CIN outcomes within the LBC 'abnormal' group (Table 9, purple boxes). For example, there were 2,344 people reported as having CIN3+ from surveillance data. According to data from Bonde *et al.*, this number represents 91.4% of people with the true disease state CIN3+, it is then possible to infer that there are a further 220 people with CIN3+ who (incorrectly) received a 'normal' LBC result and the total number of people with CIN3+ is the sum of these (n=2564).

Combining the number of LBC normal and abnormal for each CIN category then gives us the true disease prevalence of each category within the screening population (Table 9, blue boxes).

Table S9. Calculating true disease states at routine screen using LBC performance data and CIN outcomes reported in the cervical screening programme for clinician collected samples using Bonde *et al.* [23]

	True disease states						Total (Screening data adjusted for LTFU)		Total (Calculated)	
**LBC outcome**	**≤CIN1**		**CIN2**		**CIN3+**					
Positive (abnormal)	4,185	49.5%<br>16.8%	1,930	22.8%<br>81.7%	2,344	27.7%<br>91.4%	8,459	100%<br>30.8%	8,459	28.3%
Negative (normal)	20,743	96.9%<br>83.2%	433	2.0%<br>18.3%	220	1.0%<br>8.6%	18,967	100%<br>69.2%	21,397	71.7%
Total	24,928		2,363		2,564		27,426	100%	29,856	

Footnote: The percentage presented at the top represents percentage for the row (denominator is row total) and the percentage presented underneath represents the percentage for the column (denominator is column total). CIN, Cervical Intra-epithelial Neoplasia; LTFU, lost to follow-up.

Gold cells: Diagnostic outcomes i.e., CIN outcomes informed by surveillance data.

Purple cells: Performance data for LBC from Bonde *et al.* [23] study

Blue cells: Calculated values using LBC performance data and number who are LBC 'abnormal'.

Calculating the true disease states at the early recall screen (period 2) was more difficult and a number of assumptions were made. The surveillance reports categorise outcomes as ≤CIN1 and CIN2+. To calculate the proportion with CIN3+, we inferred that the ratio of CIN2 and CIN3+ was the same at period 2 as at routine screen (baseline i.e., the routine screen), in this case 45.3% CIN2 and 54.8% CIN3+ (Table 10, orange cells).

At period 2, 1,973 people had an LBC abnormal result, but the diagnostic outcome was reported for only 1,134 (58%) of these. Therefore, the number with a normal result was reduced to 58% from 25,489 to 14,650.

When test performance data were initially used to calculate the number of people with a normal LBC result, using the number with an abnormal result, the total number of people with a normal result differed greatly than the observed number in the screening programme. Therefore, for period 2, the number of people with a normal LBC result in each CIN category was calculated by splitting the 15,784 people with a normal result using the proportional split seen at routine screen (Table 10, green boxes).

The total number of people within each disease state was then calculated by combining those with abnormal and normal LBC results.

Table S10. Calculating true disease states at early recall screen using LBC performance data and CIN outcomes reported in the cervical screening programme for clinician-collected samples at period 2 and using LBC performance data from Bonde *et al.* [23]

	True disease states						Total (Screening data adjusted for LTFU)	
**LBC outcome**	**≤CIN1**		**CIN2**		**CIN3+**			
Positive (abnormal)	754	66.5%<br>5.0%	172	15.1%<br>36.6%	208	18.4%<br>58.0%	1,134	100%<br>7.2%
Negative (normal)	14,203	96.9%<br>95.0%	297	2.0%<br>63.4%	151	1.0%<br>42.0%	14,650	100%<br>92.8%
Total	14,957		468		359		15,784	100%

Footnote: The percentage presented at the top represents percentage for the row (denominator is row total) and the percentage presented underneath represents the percentage for the column (denominator is column total). CIN, Cervical Intra-epithelial Neoplasia, LTFU, lost to follow-up.

Gold cells: Diagnostic outcomes i.e., CIN outcomes informed by surveillance data.

Orange cells: Informed by the percentage with CIN2 and CIN3+ at routine screen, as only CIN2+ data are available from screening surveillance data.

Green cells: Informed by the percentage with ≤CIN1, CIN2 and CIN3+ at routine screen.

Blue cells: Calculated values.

Calculating the outcomes for DNA methylation

The true disease state data informs the number within each CIN category (Table 11, blue cells). The proportion of these with either a positive or negative result from DNA methylation is then informed from the performance studies (Table 1) presented here in Table 5, purple cells). These are used to calculate the number of people in each of the remaining cells.

Table S11. Calculating anticipated CIN diagnostic outcomes at routine screen for clinician collected samples using DNA methylation performance data from Bonde *et al.* [23]

	True disease states						Total	
**Diagnostic state**	**≤CIN1**		**CIN2**		**CIN3+**			
Positive (hypermethylation)	5,414	63.5%<br>21.7%	1,103	12.9%<br>46.7%	2,014	23.6%<br>78.5%	8531	28.6%
Negative (no methylation)	19,514	91.5%<br>78.3%	1,260	5.9%<br>53.3%	550	2.6%<br>21.5%	21,325	71.4%
Total	24,928		2,363		2,564		29,856	

Footnote: The percentage presented at the top represents percentage for the row (denominator is row total) and the percentage presented underneath represents the percentage for the column (denominator is column total). CIN, Cervical Intra-epithelial Neoplasia; LTFU, lost to follow-up.

Blue cells: True disease state data from Table 9.

Purple cells: Performance data for DNA methylation from published study (Table 7).

Repeating these calculations for self-collected samples

Surveillance data are reported separately for clinician collected and self-collected samples, therefore these calculations were repeated for the self-sampling pathway using surveillance data for self-collected samples. The assumption here is that the disease states vary between people opting to self-sample and those opting for clinician collected sampling, but HPV testing, LBC and DNA methylation would have the same performance on different sample types.

Table S12. Calculating true disease states at routine screen using LBC performance data and CIN outcomes reported in the cervical screening programme for self-collected samples and using LBC performance data from Bonde *et al.* [23]

	True disease states						Total (Screening data adjusted for LTFU)		Total (Calculated)	
**LBC outcome**	**≤CIN1**		**CIN2**		**CIN3+**					
Positive (abnormal)	227	41.2%<br>16.8%	116	21.0%<br>46.7%	209	37.8%<br>91.4%	552	-<br>35.0%	552	-<br>30.2%
Negative (normal)	1,127	88.1%<br>83.2%	132	10.4%<br>53.3%	20	1.5%<br>8.6%	1,028	-<br>65.0%	1,279	-<br>69.8%
Total	1,354		248		229		1,580		1,831	

Footnote: The percentage presented at the top represents percentage for the row (denominator is row total) and the percentage presented underneath represents the percentage for the column (denominator is column total). CIN, Cervical Intra-epithelial Neoplasia, LTFU, lost to follow-up. Surveillance data reported 899 people with LBC abnormal result, of these 61.4% (n=552) had a diagnostic outcome reported. The 1,673 people with a normal result was reduced by the same proportion (to 1,028).

Table S13. Calculating true disease states at early recall screen using LBC performance data and CIN outcomes reported in the cervical screening programme for self-collected samples and using LBC performance data from Bonde *et al.* [23]

	True disease states						Total (Screening data adjusted for LTFU)	
**LBC outcome**	**≤CIN1**		**CIN2**		**CIN3+**			
Positive (abnormal)	4	40.0%<br>57.3%	2	21.4%<br>81.7%	4	38.6%<br>91.4%	10	-<br>2.6%
Negative (normal)	330	88.1%<br>80.0%	39	10.4%<br>18.3%	6	1.5%<br>8.6%	375	-<br>97.4%
Total	334		41		10		385	

Footnote: The percentage presented at the top represents percentage for the row (denominator is row total) and the percentage presented underneath represents the percentage for the column (denominator is column total). CIN Cervical Intra-epithelial Neoplasia; LTFU, lost to follow-up. Surveillance data reported 42 people with LBC abnormal result, of these 23.8% (n=10) had a diagnostic outcome reported. The 1,573 people with a normal result was reduced by the same proportion (to 375).

Table S14. Calculating anticipated CIN diagnostic outcomes at routine screen for clinician collected samples using DNA methylation performance data from Bonde *et al.* [23]

	True disease states						Total	
**Diagnostic state**	**≤CIN1**		**CIN2**		**CIN3+**			
Positive (hypermethylation)	294	49.9%<br>21.7%	116	19.7%<br>46.7%	180	30.5%<br>78.5%	589	32.2%
Negative (no methylation)	1060	85.4%<br>78.3%	132	10.7%<br>53.3%	49	4.0%<br>21.5%	1,241	67.8%
Total	1354		248		229		1,831	

Footnote: The percentage presented at the top represents percentage for the row (denominator is row total) and the percentage presented underneath represents the percentage for the column (denominator is column total). CIN, Cervical Intra-epithelial Neoplasia; LTFU, lost to follow-up.

Repeating both sets of calculations using Luttmer *et al.* [24]

Both sets of calculations, for clinician-collected and self-collected sampling were repeated using data from Luttmer *et al.* [24] (Table 8) to inform the accuracy of LBC and DNA methylation. Data not shown. The data from both sets of calculations which were used to inform the probabilities in the model are summarised in Table 15 and Table 16.

Table S15. Parameter data used in the model to inform the probability of a negative or positive result for LBC and DNA methylation pathways.

		Clinician collected samples		Self-collected samples	
		**DNA methylation**	**LBC**	**DNA methylation**	**LBC**
**Bonde *et al.***					
Routine screen	Positive result	28.6%	28.3%	32.2%	30.2%
	Negative result	71.4%	71.7%	67.8%	69.8%
Period 2	Positive result	7.2%	7.2%	2.8%	2.6%
	Negative result	92.8%	92.8%	97.2%	97.4%
**Luttmer *et al.***					
Routine screen	Positive result	46.6%	56.8%	51.0%	60.2%
	Negative result	53.4%	43.2%	49.0%	39.8%
Period 2	Positive result	5.9%	7.2%	2.2%	2.6%
	Negative result	94.1%	92.8%	97.8%	97.4%

Footnote: Routine screen refers to routine screen, period 2 to early screen for those hrHPV positive but LBC/DNA methylation negative.

Table S16. Parameter data used in the model to inform the probability of CIN outcomes for LBC and DNA methylation pathways.

		Clinician collected samples		Self-collected samples	
		DNA methylation	LBC	DNA methylation	LBC
**Bonde**					
Routine screen	≤CIN1	63.5%	49.5%	49.9%	41.2%
	CIN2	12.9%	22.8%	19.7%	21.0%
	CIN3+	23.6%	27.7%	30.5%	37.8%
Period 2	≤CIN1	75.8%	66.5%	48.9%	40.0%
	CIN2	8.6%	15.1%	20.1%	21.4%
	CIN3+	15.7%	18.4%	31.1%	38.6%
**Luttmer**					
Routine screen	≤CIN1	48.9%	49.5%	39.3%	41.2%
	CIN2	16.1%	22.8%	14.3%	21.0%
	CIN3+	35.1%	27.7%	46.4%	37.8%
Period 2	≤CIN1	66.1%	66.5%	38.1%	40.0%
	CIN2	10.7%	15.1%	14.6%	21.4%
	CIN3+	23.3%	18.4%	47.3%	38.6%

Footnote: Routine screen refers to routine screen, period 2 to early recall screen for those hrHPV positive but LBC/DNA methylation negative.

There were no data to inform the CIN outcomes at period 2 (early recall), for the DNA methylation pathway. It was not appropriate to use the sensitivity and specificity of DNA methylation to inform this, since the use of LBC performance data was not appropriate to use for the LBC pathway, (since the calculated totals for negative/positives were very different than actual totals seen in the screening population). Therefore, the proportional change in percentage of CIN2 seen at routine screen compared to in Period 2 for LBC was used to inform the change in percentage of CIN2 seen at routine screen compared to period 2 for DNA methylation. The same approach was used for CIN3+.

Section S5. Sensitivity analyses

Figure S3. DSA tornado plots of the five most impactful variables on cost per complete screen, unnecessary colposcopies, and CIN3+ diagnoses for Scenario 1 (Bonde *et al.)* and Scenario 2 (Luttmer *et al).*

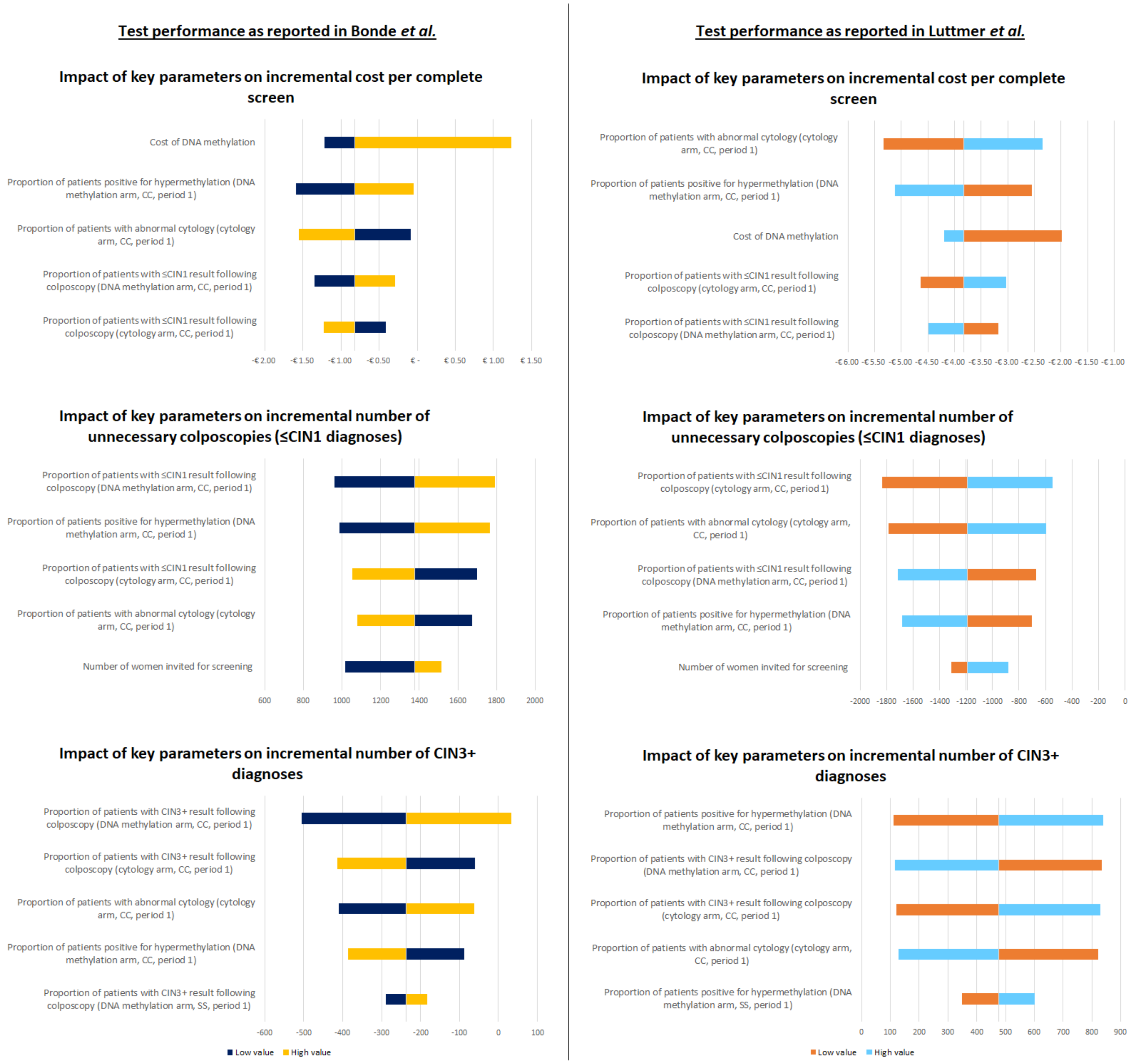

Footnote: Only the five most impactful parameters on each outcome are presented. Incremental outcomes calculated as the difference between DNA methylation and LBC outcomes (DNA methylation minus LBC). Routine screen indicated the routine screen attended. CIN, cervical intraepithelial neoplasia.

Figure S4. PSA scatter plots of the incremental cost per screen vs. incremental CIN3+ diagnoses and incremental cost per screen vs. incremental number of unnecessary colposcopies for Scenario 1 (Bonde *et al.*) and Scenario 2 (Luttmer *et al.*)

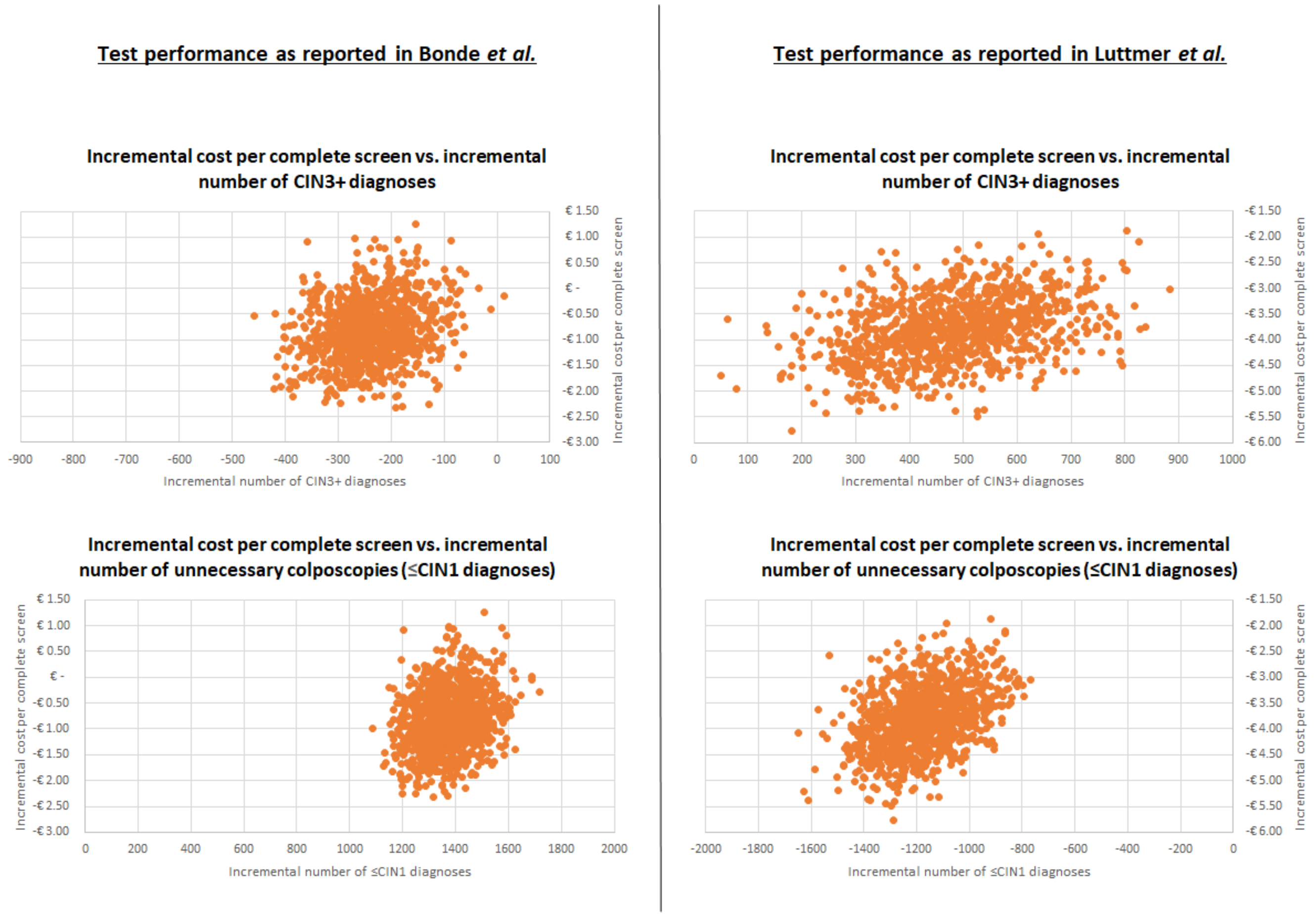

Footnote: Incremental outcomes calculated as the difference between DNA methylation and LBC outcomes (DNA methylation minus LBC). CIN, cervical intraepithelial neoplasia.

Section S6. The Dutch national cervical cancer screening programme

General Dutch cervical cancer screening pathway

Under the Dutch cervical screening programme, women between thirty and sixty years of age are invited for routine screening at five-year intervals. Subsequently, women are able to either book an appointment with general practice for a clinician-collected smear test, or request for and provide a self-collected sample through pre-paid postage. Once a sample is received, it is tested for hrHPV by one of five screening laboratories. In the absence of hrHPV in the tested sample, women are discharged to routine recall. hrHPV-positive samples are inspected using LBC for cellular abnormalities. In the case of hrHPV-positive self-collected samples, women are first required to attend a general practice appointment for a clinician-collected sample, as LBC cannot be carried out on self-collected samples. Women with normal results at LBC are recalled for a repeat screen at 6 months, while those with abnormal results are referred for colposcopy after a gynaecological appointment. Women recalled after 6 months provide a clinician-collected smear sample, which is investigated again through LBC. In the case of normal results at this stage, women are discharged to routine recall, while those with abnormal results are referred for colposcopy.

Changes to Dutch cervical cancer screening programme in 2022

Several changes were introduced in the Dutch primary cervical cancer screening programme in the Netherlands since January 2022 [6].

Eligibility criteria

Previously all women and people with a cervix aged between 30 and 60 were invited every five years for routine screening. Since 2022, the women and people with a cervix aged 40 or 50 with a negative hrHPV result in the previous screening round not invited for the following round when they are aged respectively 45 and 55. Women or people with a cervix aged 60 who had a positive hrHPV test and a normal LBC results will be invited for the next screening round when they are aged 65. Women or people with cervix aged 60 with a negative hrHPV test will not be invited for the next screening round.

Self-sampling

Previously self-sampling was only offered to women and people with a cervix who did not respond to the first two invitations to attend a smear appointment. Since January 2022, all women and people with a cervix are offered to order a self-sampling kit in the first invitation to partake in the screening programme.

Criteria for colposcopy referral

Previously all women with a HPV positive tests and a LBC result of pap2 or above were referred to colposcopy. Currently, women with HPV 16/18 and pap2 or above will be referred to colposcopy. Women who test positive for another HPV strain and pap3a2 or above will be referred to colposcopy. Women who test positive for another HPV strain and have pap2/3a1 are invited for a early recall.

Early recall period

The early recall period has been prolonged from 6 to 12 months.

Revised cervical screening pathway using DNA methylation

Incorporating DNA methylation instead of LBC as triage following hrHPV positive results does not significantly alter the screening pathway. For clinician-collected samples, the pathway remains the same, with hrHPV positive samples tested for DNA hypermethylation rather than cellular abnormalities using LBC. In the case of self-collected samples, women with positive hrHPV results are no longer required to attend a smear appointment, as DNA methylation testing can be carried out on self-collected samples.
